# The Recombinant *E*g.P29-Mediated miR-126a-5p Promotes the Differentiation of Mouse Naive CD4^+^ T Cells *via* DLK1-Mediated Notch1 Signal Pathway

**DOI:** 10.3389/fimmu.2022.773276

**Published:** 2022-02-08

**Authors:** Xiancai Du, Mingxing Zhu, Tingrui Zhang, Chan Wang, Jia Tao, Songhao Yang, Yazhou Zhu, Wei Zhao

**Affiliations:** ^1^ School of Basic Medical Science of Ningxia Medical University, Yinchuan, China; ^2^ Ningxia Key Laboratory of Prevention and Control of Common Infectious Diseases, Yinchuan, China; ^3^ Center of Scientific Technology of Ningxia Medical University, Yinchuan, China

**Keywords:** miR-126a-5p, DLK1, Notch1, CD4^+^ T cells, r*E*g.P29, Th1, Th2, *Cystic Echinococcocosis*

## Abstract

*Cystic echinococcosis* (CE) is a zoonotic parasitic disease spread worldwide caused by *Echinococcus granulosus* (*E*g), which sometimes causes serious damage; however, in many cases, people are not aware that they are infected. A number of recombinant vaccines based on *E*g are used to evaluate their effectiveness against the infection. Our previous report showed that recombinant *E*g.P29 (r*E*g.P29) has a marvelous immunoprotection and can induce Th1 immune response. Furthermore, data of miRNA microarray in mice spleen CD4^+^ T cells showed that miR-126a-5p was significantly elevated 1 week after immunization by using r*E*g.P29. Therefore, in this perspective, we discussed the role of miR-126a-5p in the differentiation of naive CD4^+^ T cells into Th1/Th2 under r*E*g.P29 immunization and determined the mechanisms associated with delta-like 1 homolog (DLK1) and Notch1 signaling pathway. One week after P29 immunization of mice, we found that miR-126a-5p was significantly increased and DLK1 expression was decreased, while Notch1 pathway activation was enhanced and Th1 response was significantly stronger. The identical conclusion was obtained by overexpression of mmu-miR-126a-5p in primary naive CD4^+^ T cells in mice. Intriguingly, mmu-miR-126a-5p was significantly raised in serum from mice infected with protoscolex in the early stages of infection and markedly declined in the late stages of infection, while has-miR-126-5p expression was dramatically reduced in serum from CE patients. Taken together, we show that miR-126a-5p functions as a positive regulator of Notch1-mediated differentiation of CD4^+^ T cells into Th1 through downregulating DLK1 *in vivo* and *in vitro*. Hsa-miR-126-5p is potentially a very promising diagnostic biomarker for CE.

## Introduction

Cystic echinococcosis (CE) is a chronic and easily neglected complicated zoonotic parasitic disease that is prevalent mainly in areas with developed animal husbandry (1, 2). It is important to note that the early stages of CE do not cause any obvious symptoms and can coexist with the host for a long period of time. The onset of clinical symptoms usually means that the cyst has grown to the point of causing damage to the parasitic site (3–5). Imaging, the conventional means of diagnosing CE, also is unable to make an effective detection of infection in its early stages (6, 7). Although there are different treatment methods such as percutaneous treatment, surgical treatment, medication, observation, and waiting at different stages of CE (8–11), it is essential to find ways to prevent the occurrence of CE and means to detect it at an early stage. Moreover, the development of vaccine is a feasible method to prevent the prevalence of CE. Several candidate vaccine molecules have been shown to be highly protective against *Echinococcus granulosus* (*E*g) infection in mice and livestock (12–17). As a representative vaccine, r*E*g.P29 showed 94.5% and 96.6% immune protection in sheep and mice, respectively (15, 18).

The P29 is encoded by a single gene in the *Echinococcus* genus and has been applied as a potential serological marker for the post-treatment monitoring of CE (19). Consistent with previous descriptions of other vaccines, the protection produced by r*E*g.P29 was dominated by a Th1 response (20–22). However, the underlying mechanism of r*E*g.P29 has remained ambiguous. In this context, this study aims to investigate the molecules and related signaling pathways by which r*E*g.P29 regulates CD4^+^ T-cell differentiation. We sequenced miRNAs from splenic CD4^+^ T cells at different time points in r*E*g.P29-immunized and protoscolex-infected mice. The results showed that miR-126a-5p, miR-378a-5p, and miR-1247-5p may be involved in the differentiation of CD4^+^ T cells toward Th1 to protect the organism from *E*g infection.

MicroRNAs (miRNAs) are a class of small non-coding RNAs that play negative regulatory effects mainly by binding to the 3′-UTR of target mRNAs (23, 24). Although there are some reports about miRNAs in parasitic diseases, studies on miRNAs in CE are little. Most studies have either focused on identifying miRNAs in different stages of the parasite or on screening for differential miRNAs in the host (25, 26). In the present study, we investigated miR-126a-5p that was upregulated after 1 week of r*E*g.P29 immunization and protoscolex infection and downregulated after 24 weeks of protoscolex infection. This observation reveals a new mechanism that miR-126a-5p enhances Notch1 signal by targeting delta-like 1 homolog (DLK1) and then promotes the differentiation of CD4^+^ T cells toward Th1. As a highly conserved signal pathway, Notch is still controversial in regulating CD4^+^ T-cell differentiation (27–31), but our results show that Notch1 can regulate CD4^+^ T-cell differentiation toward Th1.

Here, we confirmed that miR-126a-5p promotes Th1 polarization by negatively regulating DLK1, enhancing Notch1 signaling under r*E*g.P29 immune induction, and that miR-126-5p could be a very promising biomarker for CE.

## Materials and Methods

### Ethics Statement

Written informed consent was requested from the patients and this study was reviewed and approved by the Ethics Committee of Ningxia Medical University. Meanwhile, the study on experimental animals was also reviewed and authorized by the Ethics Committee of Ningxia Medical University (Approval No. 2018-078). The data acquired were consistent with relevant ethical requirements.

### Animals

Female BALB/C mice at 6–8 weeks weighing 18–25 g were purchased from the Experimental Animal Center of Ningxia Medical University. All mice were placed in a specific pathogen-free (SPF) environment at 22°C. Mice studies and euthanasia procedures were performed in accordance with the Ningxia Medical University Animal Welfare Guidelines.

### Expression and Purification of r*E*g.P29 Protein

Our laboratory previously constructed and recombined the *E*g.P29 gene from *E. granulosus* (GenBank ID: AF078931) in *Escherichia coli* by using the pET28a plasmid (15, 18). The strain expressing r*E*g.P29 was inoculated in LB solid medium and incubated at 37°C in an inverted position. Monoclonal colonies were picked into LB liquid medium (containing kanamycin) at 37°C in a constant temperature shaker and incubated at 200 rpm overnight. When the bacterial broth appeared turbid, the broth and medium were diluted 1:100 and continued to expand the culture to log phase, followed by the addition of IPTG to induce r*E*g.P29 protein expression. The strains obtained after centrifugation were treated with lysozyme and phenylmethanesulfonyl fluoride, and subsequently, the strains were lysed under ultrasound. The purified r*E*g.P29 was obtained after equilibration and binding columns according to the protocol of the manufacturer. The r*E*g.P29 was used for immunization after endotoxin was removed.

### Immunization and Infection of BALB/c Mice

For the r*E*g.P29 immunization, 21 female BALB/c mice at 6–8 weeks were randomly divided into three groups (PBS group, FCA group, and r*E*g.P29+FCA group) with seven mice in each group. The mice were immunized by abdominal subcutaneous three-point injection. Each mouse in the PBS group was injected with 100 μl PBS (phosphate buffered saline), each mouse in the FCA group was injected with 20 μg FCA (Freund’s complete adjuvant) diluted with 100 μl PBS, and each mouse in the r*E*g.P29+FCA group was injected with 100 μl PBS diluted with 20 μg r*E*g.P29 and 20 μg FCA. For the protoscolex infection, the protoscolex was isolated from the cyst of a CE patient at the Department of Hepatobiliary Surgery, General Hospital of Ningxia Medical University. The 10 female BALB/c mice at 6–8 weeks were randomly divided into two groups (PBS group and infected group). Each mouse in the infected group was injected intraperitoneally with 200 μl of diluted protoscolex (approximately 2,000), and each mouse in the PBS group was injected intraperitoneally with the same 200 μl PBS.

### Isolation of CD4^+^ T Cells and Naive CD4^+^ T Cells

Mice were euthanized by cervical dislocation and soaked into 75% alcohol, and sterile spleens were obtained by cutting off the epidermis. Lymphocytes were isolated from the spleen with the mouse spleen lymphocyte cell separation medium kit (Tianjin HaoYang Biological Manufacture Co., Ltd, China). The CD4^+^ T cells were purified from lymphocytes by the mouse CD4^+^ T cell isolation kit (Miltenyi Biotec, Inc, Cologne, Germany). The naive CD4^+^ T cells were purified from the mouse naive CD4^+^ T cell isolation kit (Miltenyi Biotec, Inc, Cologne, Germany).

### Culture of Cells

The naive CD4^+^ T cells were cultured in 1640 medium (Solarbio Science & Technology Co., Ltd, Beijing, China) containing 10% serum (Gibco, Thermo Fisher Scientific Co., Ltd, Xian, China) and 1% penicillin–streptomycin (Solarbio Science & Technology Co., Ltd, Beijing, China.) and placed in an incubator at 37°C and 5% CO_2_. HEK293T cells came from laboratory conservation. After resuscitation, HEK293T cells were cultured in DMEM medium (Solarbio Science & Technology Co., Ltd, Beijing, China.) containing 10% serum and 1% penicillin–streptomycin and placed in an incubator at 37°C and 5% CO_2_.

### Differentiation of Naive CD4^+^ T Cells

The naive CD4^+^ T cells were cultured in 96-well plates with 1 × 10^5^ cells, in 24-well plates with 5 × 10^5^ cells, or in 6-well plates with 5 × 10^6^ cells (precoated with anti-CD3, 1 μg/ml, Thermo Fisher Scientific Co., Ltd, Xian, China) and were then transfected with miR-126a-5p mimics (60 nM, QIAGEN, Germany) and inhibitor (150 nM, QIAGEN, Germany) using HiPerFect transfection reagent (QIAGEN, Germany) or DLK1-siRNA (56 nM, Shanghai GenePharma Co., Ltd, China) using Lip3000 (Shanghai GenePharma Co., Ltd, China) or OE-DLK1 using polybrene (Solarbio Science & Technology Co., Ltd, Beijing, China) or DAPT (γ-secretase inhibitor, 20 μM, Sigma-Aldrich LLC). After 4 h, soluble anti-CD28 (0.2 μg/ml, Thermo Fisher Scientific Co., Ltd, Xian, China) was added along with cytokines that differentiate naive CD4^+^ T cells into different T-cell subtypes. For Th1 cells, IL-2 (20 ng/ml, BioLegend, Inc, California, USA.), IL-12 (50 ng/ml, BioLegend, Inc, California, USA.), and anti-IL-4 antibody (10 ng/ml, BioLegend, Inc.) were added to the well plates for 72 h. To differentiate the cells into Th2 cells, IL-2 (20 ng/ml, BioLegend, Inc, California, USA.), IL-4 (10 ng/ml, BioLegend, Inc, California, USA.), and anti-IL-IFN-γ antibody (10 ng/ml, BioLegend, Inc, California, USA.) were added to the well plates for 72 h.

### Staining and Flow Cytometry

In order to detect the expression of IFN-γ and IL-4 in transfected naive CD4^+^ T cells after immunization, the cells were surface stained with PerCP-Cyanine5.5-anti-CD3 (Thermo Fisher Scientific Co., Ltd, Xian, China) and APC-anti-CD4 antibodies (Thermo Fisher Scientific Co., Ltd, Xian, China). Next, the cells were treated with fixation buffer (BioLegend, Inc, California, USA.) and permeabilization buffer (BioLegend, Inc, California, USA.) followed by intracellular staining with fluorochrome-conjugated PE-anti-IFN-γ (Thermo Fisher Scientific Co., Ltd, Xian, China) and PE-anti-IL-4 (Thermo Fisher Scientific Co., Ltd, Xian, China). When assaying CD4^+^ T cells from immunized mice, staining is performed using PerCP-Cyanine5.5-anti-CD3 (Thermo Fisher Scientific Co., Ltd, Xian, China), APC-anti-CD4 antibodies (Thermo Fisher Scientific Co., Ltd, Xian, China), PE-anti-IFN-γ (Thermo Fisher Scientific Co., Ltd, Xian, China), and BV605-anti-IL-4 (Thermo Fisher Scientific Co., Ltd, Xian, China). The degree of cell activation after transfection has been determined by staining the surface of naive CD4^+^ T cells with PE-anti-CD25 and FITC-anti-CD69 antibodies (Thermo Fisher Scientific Co., Ltd, Xian, China). The stained cells were measured by a BD FACSCelesta instrument (Beijing, China) and the results were analyzed by FlowJo.

### ELISA

After transfection and cytokine stimulation, naive CD4^+^ T cells were cultured for 72 h and the supernatant was collected by centrifugation. The secreted cytokines IFN-γ, IL-2, TNF-α, IL-4, IL-5, and IL-10 were measured with ELISA kits (Jiangsu Meimian industrial Co., Ltd, China.) according to the protocol of the manufacturer. IFN-γ, IL-2, and TNF-α are representative factors for Th1 cells. IL-4, IL-5, and IL-10 are characteristic factors for Th2 cells.

### qRT-PCR

The total RNA was extracted from CD4^+^ T cells/naive CD4^+^ T cells with TRIzol reagent (Thermo Fisher Scientific Co., Ltd, Xian, China.). For mRNA reverse transcription, cDNA synthesis was performed with the First Strand Synthesis - 1st Strand cDNA Synthesis Kit (Thermo Fisher Scientific Co., Ltd, Xian, China.), and for specific miRNAs, reverse transcription was obtained with the Mir-X miRNA First-Strand Synthesis Kit (Takara Biomedical Technology Co., Ltd, Beijing, China.). The gene expression of mRNA and miRNA was performed on ABI 7500 fast real-time PCR system (Thermo Fisher Scientific Co., Ltd, Xian, China.) with Bestar^®^ Sybr Green qPCR Master Mix (DBI^®^ Bioscience) and Mir-X miRNA qRT-PCR TB Green^®^ Kit (Takara Biomedical Technology Co., Ltd, Beijing, China.), respectively. U6 and GAPDH were used as endogenous references, and the 2^−ΔΔCT^ method was used to calculate the gene expression level. The primer sequence details are listed in **Table 1**.

**Table 1:** Sequences of primers involved in qRT-PCR.

Gene	Forward primer (5′–3′)	Reverse primer (5′–3′)
*miR-126a-5p*	CATTATTACTTTTGGTACGCG	GCAGGGTCCGAGGTATTC
*U6*	GCTTCGGCAGCACATATACTAA	CGAATTTGCGTGTCATCCTT
*IFN-y*	GCCACGGCACAGTCATTGA	TGCTGATGGCCTGATTGTCTT
*IL-4*	ATCATCGGCATTTTGAACGAGG	TGCAGCTCCATGAGAACACTA
*T-bet*	CTTGGATCCTTCGCCTACCC	CTTCCCAGACACCTCCAACC
*GATA-3*	GAGAGCGAGACATAGAGAGC	AGACGGTTGCTCTTCCGATC
*HESl*	GCCAATTTGCCTTTCTCATC	AGGTGACACTGCGTTAGG
*Notchl*	AGTGGACATTGACGAGTG	GGCATAAGCAGAGGTAGG
*DLKl*	CCATCTGCTTCACCATCC	CTCGCCGCTGTTATACTG
*GAPDH*	GGTTGTCTCCTGCGACTTCA	TGGTCCAGGGTTTCTTACTCC

### Western Blot

Total protein was extracted from treated cells with whole cell lysis assay (KeyGen Biotech Co., Ltd, Jiangsu, China). The concentration of protein was determined with BCA protein quantitation assay (KeyGen Biotech Co., Ltd, Jiangsu, China). The 5× SDS-PAGE loading buffer (Solarbio Science & Technology Co., Ltd, Beijing, China.) was added to the protein solution and denatured in metal bath at 100°C for 8 min. The treated protein was separated by 12% SDS-PAGE and transferred to 0.2 μM PVDF membrane and closed by PBST of 5% skimmed milk for 2 h at room temperature. Then, the primary antibodies were incubated overnight at 4°C, and after washing the membrane with PBST, the secondary rabbit anti-mouse antibodies (Cell Signaling Technology, Inc, Danvers, Massachusetts, USA.) were incubated for 2 h at room temperature and finally protein expression assays were performed on ChemiDoc™ Touch Imaging System (Bio-Rad Laboratories, Inc, Shanghai, China) with ECL detection kit (KeyGen Biotech Co., Ltd, Jiangsu, China.). β-Actin was used as an endogenous reference. The primary antibodies were Ab against HES1, HE5, Notch1, N1ICD (Abcam Plc, Shanghai, China.), and actin (Cell Signaling Technology, Inc, Danvers, Massachusetts, USA.).

### Dual-Luciferase Reporter Assay

The luciferase-3′-UTR reporter system was used to verify the direct relationship of DLK1 transcripts with miR-126a-5p. The complete segment of DLK1 3′-UTR that is located downstream of the Renilla luciferase coding sequence (*Xho*I/*Not*I site) was cloned in the psiCheck-2 plasmid (Promega, Shanghai, China.). It was subsequently co-transfected with miR-126a-5p mimics or NC (50 ng) into HEK293T cells. The cells were then incubated at 37°C for 48 h and collected in Dual-Glo dual luciferase assay system (Promega, Shanghai, China.) to measure Firefly and Renilla luciferase activity. For each sample, the Firefly luciferase activity was normalized to the expression of Renilla luciferase activity.

### CFSE Assay

To characterize the proliferation of transfected cells, the concentration of isolated naive CD4^+^ T cells was adjusted to 1 × 10^7^/ml. CFSE (2.5 μM, Thermo Fisher Scientific Co., Ltd, Xian, China.) was added to 1 ml of cell suspension and incubated for 15 min at 37°C and protected from light. Then, precooled 1640 medium containing 40% FBS was added and incubated at 4°C for 5 min away from light to terminate the staining. After termination of staining, the cells were washed by centrifugation and resuspended with complete medium. Finally, the cells treated with miR-126a-5p or DLK1 or DAPT were assayed for proliferation level by BD FACSCelesta instrument.

### Collection of the Serum Sample

Peripheral blood of hepatic CE patients (*n* = 12) was collected from the Department of Hepatobiliary Surgery, General Hospital of Ningxia Medical University, and healthy controls (*n* = 16) were obtained from the population in Ningxia Province from June 2018 to June 2021. Patients with concurrent tumors, autoimmune diseases, or other infectious diseases were excluded. All patients were diagnosed with CE by imaging, supplemented by serology. According to the World Health Organization (WHO) ultrasound classification criteria for hydatid cyst of the liver (32), three patients in this study had stage CE1, five had stage CE2, and four had stage CE4. The CE1 and CE2 phases are active, CE3 is transitional, and CE4 and CE5 are inactive. The patients had hepatic cysts in the right lobe of the liver in nine cases and in the left lobe of the liver in three cases. Healthy control subjects were of similar age and, like CE patients, free of tumors, autoimmune diseases, and other infectious diseases and seronegative. All study subjects provided informed consent. The general information of the included study subjects is shown in **Table 2**. One and 24 weeks after r*E*g.P29 immunization of mice, peripheral blood was collected. Similarly, peripheral blood was collected from mice infected with protoscolex for 1 and 24 weeks. The samples were allowed to clot at room temperature for 1 h, then centrifuged at 2,000*g* for 5 min, and the supernatant was the serum.

**Table 2:** Characteristics of the study subjects.

Group	CE (*n* = l2)	HC (*n* = l6)
Age	44.67 ± 6.7	43.3 ± 4.6
Gender		
Male	7 (58.33%)	7 (43.75%)
Female	5 (41.67%)	9 (56.25%)
Cyst stage
	CEl (*n* = 3)	–
	CE2(*n* = 5)	–
	CE4 (*n* = 4)	–
Localization	Liver right lobe (*n* = 9)	–
	Liver left lobe (*n* = 3)	–

Values for age are expressed as mean ± standard deviation.

CE, cystic echinococcosis; HC, healthy controls; –, not determined.

### Statistical Analysis

All data were analyzed and processed as mean ± SD with GraphPad Prism software 7.0 (GraphPad Software). Student’s *t*-test was used to analyze the data of two groups, while one-way ANOVA was used to analyze the data of multiple groups. The results were considered statistically significant at *P <*0.05.

## Results

### Upregulation of miR-126a-5p Expression in Splenic CD4^+^ T Cells 1 Week After r*E*g.P29 Immunization in Mice

**Figure 1:**
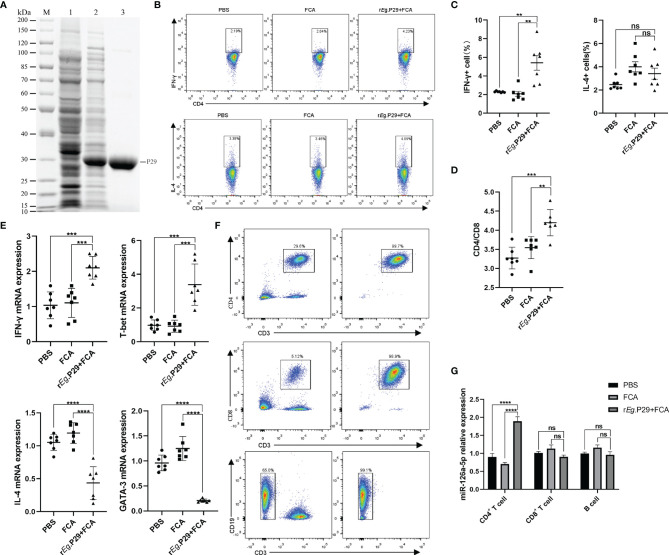
Upregulation of miR-126a-5p expression in splenic CD4+ T cells 1 week after rEg.P29 immunization in mice. **(A)** rEg.P29 protein in SDS-PAGE: M, protein markers; line 1, Escherichia coli lysates; line 2, induction of E. coli lysates with IPTG; line 3, purified rEg.P29. **(B)** Flow assay of IFN-g and IL-4 expression levels in splenic CD4+ T cells 1 week after rEg.P29 immunization. **(C)** Statistical graph of flow analysis of IFN-g and IL-4. **(D)** Ratio of splenic CD4+T cells to CD8+ T cells 1 week after rEg.P29 immunization. **(E)** Expression levels of IFN-g, T-bet, IL-4, and GATA-3 in splenic CD4+ T cells after 1 week of rEg.P29 immunization by qRT-PCR. **(F)** Flow sorting of CD4+ T cells, CD8+ T cells, and B cells from spleen lymphocytes. **(G)** Expression of miR-126a-5p in CD4+ T cells, CD8+ T cells, and B cells was detected by qRT-PCR. **P<0.01, ***P < 0.001, ****P < 0.0001; ns, not significant.

The r*E*g.P29 strain preserved in the laboratory was identified by protein sequencing to ensure that no mutations were present under BLAST comparison. With this in mind, purified r*E*g.P29 was used to immunize mice after endotoxin removal (**Figure 1A**). One week after immunization of mice with r*E*g.P29, the results of flow cytometry showed significantly higher IFN-γ expression producing the Th1 subtype and no significant change in IL-4 expression producing the Th2 subtype in splenic CD4^+^ T cells compared with the FCA and PBS groups (**Figures 1B, C**), which is consistent with previous descriptions (20). The ratio of CD4^+^ T cells to CD8^+^ T cells was also upregulated in the r*E*g.P29 group (**Figure 1D**). Interestingly, qRT-PCR results in CD4^+^ T cells showed an elevated expression of IFN-γ and T-bet, while IL-4 and GATA-3 expression was decreased (**Figure 1E**). We then purified CD4^+^ T cells, CD8^+^ T cells, and B cells from different groups of mouse spleen lymphocytes (**Figure 1F**) and examined the miR-126a-5p expression levels in different cells, respectively. The qRT-PCR results showed that miR-126a-5p expression was significantly higher in CD4^+^ T cells in the r*E*g.P29 group compared with the PBS and FCA groups. miR-126a-5p expression was not different in CD8^+^ T cells and B cells (**Figure 1G**). In summary, r*E*g.P29 promoted the expression of miR-126a-5p in spleen CD4^+^ T cells of mice 1 week after immunization. miR-126a-5p may be involved in the differentiation of Th1.

### miR-126a-5p Promotes the Differentiation of Naive CD4^+^ T Cells Into the Th1 Subset

**Figure 2:**
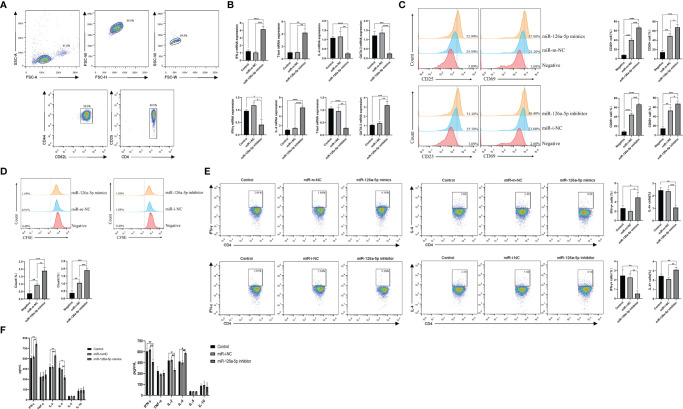
miR-126a-5p promotes the differentiation of naive CD4^+^ T cells into Th1. **(A)** The purification rate of naive CD4^+^ T cells from lymphocytes sorted by magnetic beads with flow cytometry. **(B)** Expression of IFN-γ, T-bet, IL-4, and GATA-3 in naive CD4^+^ T cells transfected with miR-126a-5p mimics or miR-126a-5p inhibitor was examined by qRT-PCR. **(C)** Flow cytometry determination of activation indicators CD25 and CD69 after transfection with miR-126a-5p mimics or miR-126a-5p inhibitor in naive CD4^+^ T cells. **(D)** CFSE assay of the proliferation levels in naive CD4^+^ T cells after transfection with miR-126a-5p mimics or miR-126a-5p inhibitor. **(E)** Flow cytometry measurement of IFN-γ and IL-4 expression after transfection with miR-126a-5p mimics or miR-126a-5p inhibitor in naive CD4^+^ T cells. **(F)** ELISA detection of IFN-γ, TFN-α, IL-2, IL-4, IL-5, and IL-10 expression in culture supernatants after transfection with miR-126a-5p mimics or miR-126a-5p inhibitor in naive CD4^+^ T cells. *P < 0.05, **P < 0.01, ***P < 0.001, ****P < 0.0001.

To investigate the role of miR-126a-5p in CD4^+^ T cells, we first used magnetic beads to sort spleen naive CD4^+^ T cells from BALB/C mice, with a purity of more than 99% (**Figure 2A**). The effect of miR-126a-5p on T-cell differentiation was defined by transfection with miR-126a-5p mimics and miR-126a-5p inhibitor. The most suitable concentrations for miR-126a-5p mimics and inhibitor were 60 and 150 nM (**Supplementary Figures 1A, B**), respectively, so subsequent experiments were performed at these concentrations. The purified naive CD4^+^ T cells were transfected with miR-126a-5p mimics or inhibitor and cultured under anti-CD3 and anti-CD28 as well as cytokines required for differentiation to Th1 and Th2. The qRT-PCR analysis after coculture for 72 h showed that the expression of IFN-γ and T-bet increased while IL-4 and GATA-3 decreased compared with control (naive CD4^+^ T cells with anti-CD3, anti-CD8, and cytokines) and miR-m-NC groups (miR-126a-5p mimics negative control, naive CD4^+^ T cells with anti-CD3, anti-CD8, cytokines, and control miRNA sequence). Furthermore, compared with control and miR-i-NC (miR-126a-5p inhibitor negative control, naive CD4^+^ T cells with anti-CD3, anti-CD8, cytokines, and control miRNA sequence), the results were reversed for transfected miR-126a-5p inhibitor (**Figure 2B**). Flow analysis of naive CD4^+^ T-cell activation status under anti-CD3 and anti-CD4 stimulation by surface staining with anti-CD25 and anti-CD69 showed that either miR-126a-5p mimics or miR-126a-5p inhibitor was able to promote naive CD4^+^ T-cell activation compared with negative (naive CD4^+^ T cells without anti-CD3, anti-CD8) and related control (**Figure 2C**). By the same token, either miR-126a-5p mimics or miR-126a-5p inhibitor can promote CD4^+^ T-cell proliferation by CFSE (**Figure 2D**). Despite the effect on both activation and proliferation of naive CD4^+^ T cells, the flow analysis results showed a positive correlation between IFN-γ and miR-126a-5p expression and a negative correlation between IL-4 and miR-126a-5p expression (**Figure 2E**). The ELISA of supernatants from the above cell cultures showed that IFN-γ and IL-2 were highly expressed and IL-4 was low in the miR-126a-5p mimics group. The miR-126a-5p inhibitor group showed the opposite expression of cytokines, while TNF-α, IL-5, and IL-10 were not significantly different in both groups (**Figure 2F**). Overall, the experiments confirmed that miR-126a-5p promotes the differentiation of naive CD4^+^ T cells toward Th1.

### miR-126a-5p Regulates the Notch1 Signaling Pathway by Targeting DLK1

**Figure 3:**
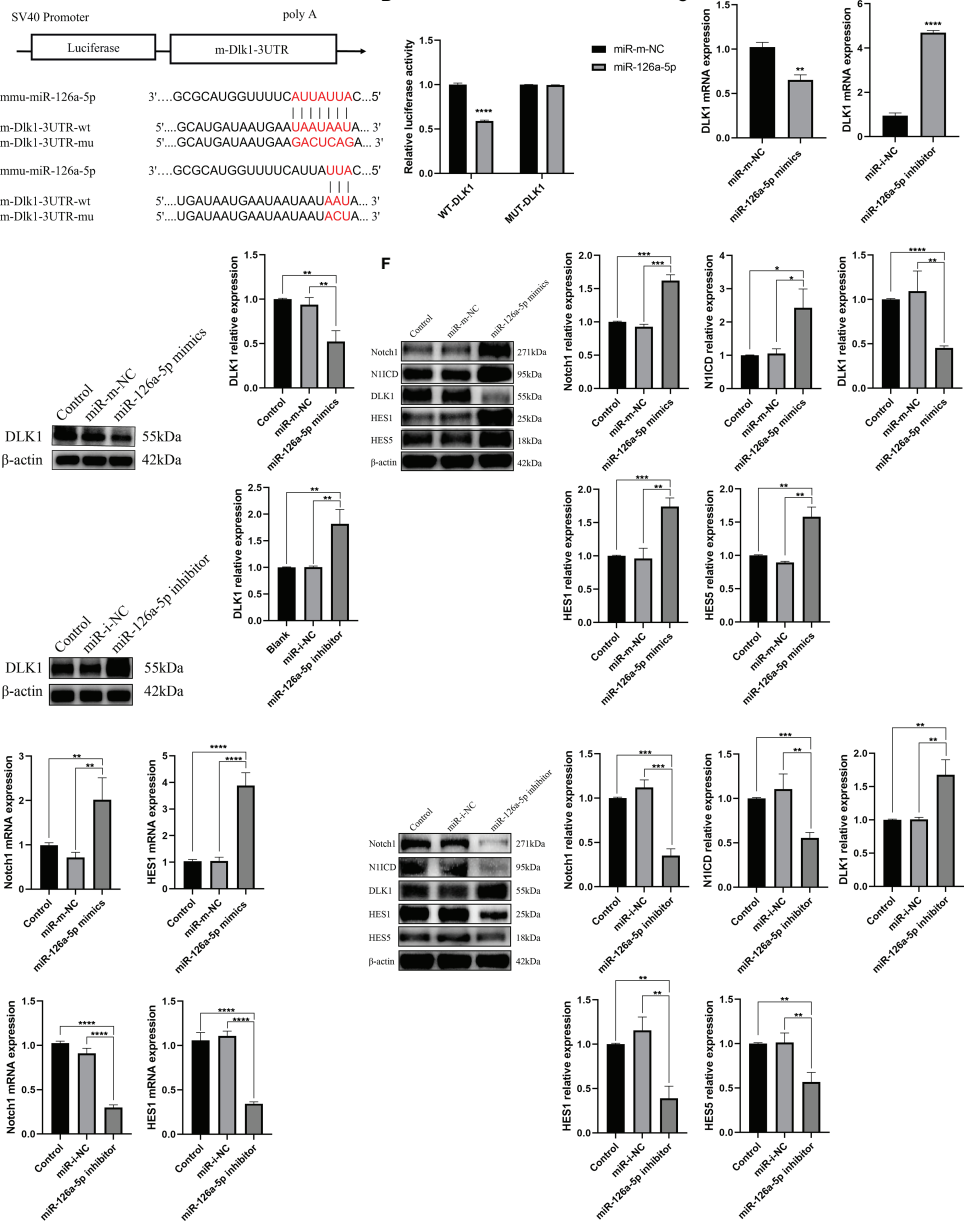
miR-126a-5p regulates the Notch1 signaling pathway by targeting DLK1. **(A)** Predicted binding site of miR-126a-5p on DLK1. **(B)** The luciferase activity of mir-126a-5p binding to DLK1 was measured by dual luciferase reporter gene. **(C)** DLK1 mRNA expression after transfection with miR-126a-5p mimics or miR-126a-5p inhibitor in naive CD4^+^ T cells determined by qRT-PCR. **(D)** DLK1 protein expression after transfection with miR-126a-5p mimics or miR-126a-5p inhibitor in naive CD4^+^ T cells by Western blot. **(E)** Notch1 and HES1 mRNA expression after transfection with miR-126a-5p mimics or miR-126a-5p inhibitor in naive CD4^+^ T cells determined by qRT-PCR. **(F)** The protein expression of Notch1, N1ICD, DLK1, HES1, and HES5 after transfection with miR-126a-5p mimics or miR-126a-5p inhibitor in naive CD4^+^ T cells by Western blot. *P < 0.05, **P < 0.01, ***P < 0.001, ****P < 0.0001.

It was reported that miR-126a-5p plays an important role in limiting atherosclerosis by targeting DLK1 (33). However, to date, the relevance of the miR-126a-5p, DLK1, and differentiation of CD4^+^ T cells has not been investigated. In this study, we analyzed miR-126a-5p with binding sites to DLK1 by a combination of three sites, TargetScan, miRDB, and DIANA (**Figure 3A**). Dual luciferase results showed that co-transfection of miR-126a-5p mimics in 293T cells significantly inhibited the luciferase activity of DLK1 wild-type 3′-UTR (**Figure 3B**). The qRT-PCR and Western blot assays also showed a significant inhibitory effect of miR-126a-5p on the expression of DLK1 in naive CD4^+^ T cells (**Figures 3C, D**). DLK1, as a homolog of the ligand DLL, competitively binds to the Notch1 receptor to affect the Notch1 signaling pathway (34–36). Alternatively, accumulated evidence indicates that Notch1 signaling pathway was involved in CD4^+^ T-cell differentiation into the Th1 subtype (29, 37, 38). We then examined the effect of miR-126a-5p on the Notch1 signaling pathway in naive CD4^+^ T cells. The qRT-PCR and Western blot data showed that Notch1 signaling maintained the same expression trend as miR-126a-5p (**Figures 3E, F**). The expression of Notch1, N1ICD, HES1, and HES5 increased with the elevation of miR-126a-5p. The above findings indicate that miR-126a-5p can promote Notch1 signaling pathway through targeted inhibition of DLK1.

### DLK1 Modulates Naive CD4^+^ T-Cell Differentiation Through the Notch1 Signaling Pathway

**Figure 4:**
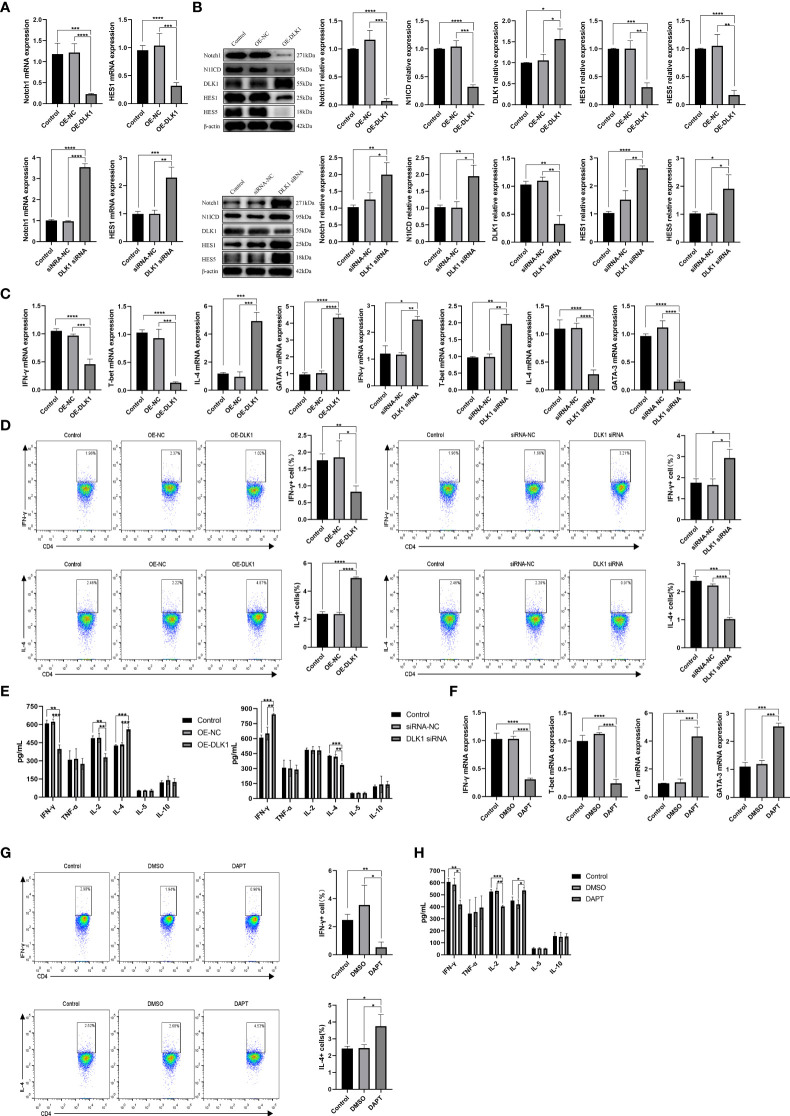
DLK1 modulates naive CD4^+^ T-cell differentiation through the Notch1 signaling pathway. **(A)** Notch1 and HES1 mRNA expression after transfection with OE-DLK1 or DLK1 siRNA in naive CD4^+^ T cells determined by qRT-PCR. **(B)** The protein expression of Notch1, N1ICD, DLK1, HES1, and HES5 after transfection with OE-DLK1 or DLK1 siRNA in naive CD4^+^ T cells by Western blot. **(C)** qRT-PCR detection of mRNA expression of IFN-γ, T-bet, IL-4, and GATA-3 after transfection with OE-DLK1 or DLK1 siRNA in naive CD4^+^ T cells. **(D)** Expression of IFN-γ and IL-4 after transfection with OE-DLK1 or DLK1 siRNA in naive CD4^+^ T cells determined by flow cytometry. **(E)** The expression of IFN-γ, TNF-α, IL-2, IL-4, IL-5, and IL-10 after transfection with OE-DLK1 or DLK1 siRNA in naive CD4^+^ T cells measured by ELISA. **(F)** qRT-PCR detection of mRNA expression of IFN-γ, T-bet, IL-4, and GATA-3 after transfection with DAPT in naive CD4^+^ T cells. **(G)** Expression of IFN-γ and IL-4 after transfection with DAPT in naive CD4^+^ T cells determined by flow cytometry. **(H)** The expression of IFN-γ, TNF-α, IL-2, IL-4, IL-5, and IL-10 after transfection with DAPT in naive CD4^+^ T cells measured by ELISA. *P < 0.05, **P < 0.01, ***P < 0.001, ****P < 0.0001.

We demonstrated the effect of DLK1 on the Notch1 signaling pathway and naive CD4^+^ T-cell differentiation by transfecting lentivirus-overexpressing DLK1 (OE-DLK1) or DLK1 siRNA (**Supplementary Figure 2A**). The DLK1 siRNA-523 fragment was the most effective, and the following experiments were performed with DLK1 siRNA-523, referred to as DLK1 siRNA. Compared with OE-NC (OE-negative control), the OE-DLK1 group resulted in impaired Notch1 pathway and reduced the expression of HES1, HES5, N1ICD, and Notch at 48 h post-transfection. The opposite results were observed in the transfected DLK1 siRNA group (**Figures 4A, B**). Curiously, neither OE-DLK1 nor DLK1 siRNA promoted naive CD4^+^ T-cell activation and proliferation (**Supplementary Figures 2B, C**), but only on their differentiation. When we increased the level of DLK1 in naive CD4^+^ T cells, qRT-PCR and flow analysis showed an increase in Th2 subtypes, and ELISA data revealed an increase in secreted IL-4 and a decrease in IFN-γ and IL-2 in culture supernatants. While DLK1 levels were suppressed in naive CD4^+^ T cells, qRT-PCR, ELISA, and flow assays showed a trend toward Th1 subtypes (**Figures 4C–E**). Moreover, DAPT was used to validate the effect of impaired Notch1 signaling pathway on the differentiation of naive CD4^+^ T cells (**Supplementary Figure 2D**). Consistent with the previous findings, inhibition of the Notch1 signaling pathway did not affect the activation and proliferation of naive CD4^+^ T cells, but rather their differentiation at 48 h (**Supplementary Figures 2E, F**). Inhibited Notch1 signaling decreased the expression of IFN-γ, while IL-4 expression was elevated (**Figures 4F–H**). In general, DLK1 can negatively affect the Notch1 signaling pathway to regulate naive CD4^+^ T-cell differentiation.

### miR-126a-5p and DLK1 Together Can Rescue Naive CD4^+^ T-Cell Differentiation Induced by Their Separate Actions

**Figure 5:**
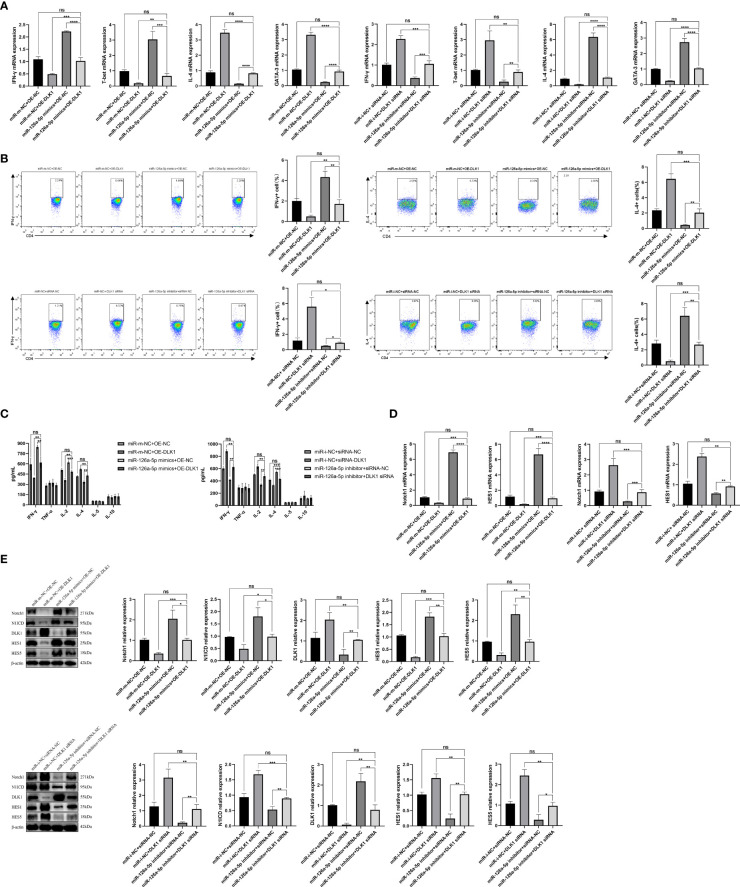
miR-126a-5p and DLK1 together can rescue the naive CD4^+^ T-cell differentiation induced by their separate actions. **(A)** The mRNA expression of IFN-γ, T-bet, IL-4, and GATA-3 after transfection with miR-126a-5p mimics+OE-DLK1 or miR-126a-5p inhibitor+DLK1 siRNA in naive CD4^+^ T cells performed by qRT-PCR. **(B)** The expression of IFN-γ and IL-4 after transfection with miR-126a-5p mimics+OE-DLK1 or miR-126a-5p inhibitor+DLK1 siRNA in naive CD4^+^ T cells conducted by flow cytometry. **(C)** The expression of IFN-γ, TNF-α, IL-2, IL-4, IL-5, and IL-10 after transfection with miR-126a-5p mimics+OE-DLK1 or miR-126a-5p inhibitor+DLK1 siRNA in naive CD4^+^ T cells measured by ELISA. **(D)** Notch1 and HES1 mRNA expression after transfection with miR-126a-5p mimics+OE-DLK1 or miR-126a-5p inhibitor+DLK1 siRNA in naive CD4^+^ T cells determined by qRT-PCR. **(E)** The protein expression of Notch1, N1ICD, DLK1, HES1, and HES5 after transfection with miR-126a-5p mimics+OE-DLK1 or miR-126a-5p inhibitor+DLK1 siRNA in naive CD4^+^ T cells by Western blot. *P < 0.05, **P < 0.01, ***P < 0.001, ****P < 0.0001; ns, not significant.

In order to further verify the effects of miR-126a-5p and DLK1 on naive CD4^+^ T-cell differentiation, we co-transfected miR-126a-5p mimics + OE-DLK1 (miR-m-NC + OE-NC as control) or miR-126a-5p inhibitor + DLK1 siRNA (miR-i-NC + siRNA-NC as control) in T cells and measured the expression of Th1/Th2 subtypes. After co-transfection into naive CD4^+^ T cells, qRT-PCR, flow analysis, and ELISA data demonstrated that either the miR-126a-5p mimics + OE-DLK1 group or the miR-126a-5p inhibitor + DLK1 siRNA group could retrace their individual effects and reach the levels of the respective miR-m-NC + OE-NC group or miR-i-NC + siRNA-NC group (**Figures 5A**–**C**). As with the results above, the combined effect was also shown by qRT-PCR and Western blot experiments to alleviate the alterations causing the Notch1 pathway (**Figures 5D, E**). Therefore, miR-126a-5p together with DLK1 can affect the Notch1 signaling pathway and naive CD4^+^ T-cell differentiation, indicating that miR-126a-5p regulates naive CD4^+^ T-cell differentiation through the DLK1/Notch1 axis.

### Reduced DLK1 Expression and Enhanced Notch1 Signaling Pathway in Spleen CD4^+^ T Cells From r*E*g.P29-Immunized Mice

**Figure 6:**
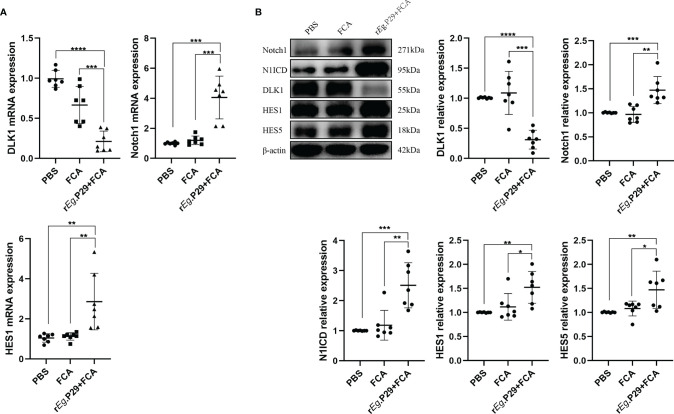
Reduced DLK1 expression and enhanced Notch1 signaling pathway in spleen CD4^+^ T cells from r*E*g.P29-immunized mice. **(A)** Identification of DLK1, Notch1, and HES1 mRNA expression in naive CD4^+^ T cells after r*E*g.P29 immunization in mice 1 week after qRT-PCR. **(B)** Western blot detection of DLK1, Notch1, N1ICD, HES1, and HES5 protein expression in CD4^+^ T cells 1 week after r*E*g.P29 immunization in mice. *P < 0.05, **P < 0.01, ***P < 0.001, ****P < 0.0001.

Although the effect of DLK1 and Notch1 on naive CD4^+^ T-cell differentiation was verified in *in-vitro* experiments, further proof is needed whether relevant changes occurred in mice 1 week after r*E*g.P29 immunization. qRT-PCR and Western blot techniques were used to characterize the expression of DLK1 and Notch1 signaling pathway-related molecules in splenic CD4^+^ T cells of mice 1 week after r*E*g.P29 immunization, compared with the PBS and FCA groups. As a target of miR-126a-5p, the expression of DLK1 was decreased, while Notch1, N1ICD, HES1, and HES5 were all elevated (**Figures 6A, B**). These data indicate that 1 week after r*E*g.P29 immunization of mice, the expression of miR-126a-5p was upregulated *in vivo*, which put the organism in a highly protective state with elevated expression of Th1 phenotype cells by reducing DLK1 expression and enhancing the Notch1 signaling pathway. Being derived from *E*g, the r*E*g.P29 is able to mimic the state in which *E*g first invades the host, resulting in immune activation of the organism and high expression of the Th1 phenotype, thus eliminating r*E*g.P29.

### Peripheral Blood Serum miR-126a-5p Was Elevated in the Early Stages of Infection With *Echinococcus granulosus* and Declined in the Late Stages of Infection

**Figure 7:**
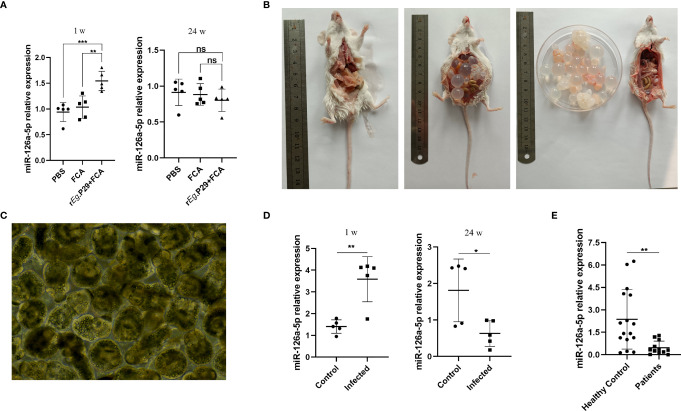
Peripheral blood serum miR-126a-5p was elevated in the early stages of infection with *Echinococcus granulosus* and declined in the late stages of infection. **(A)** Examination of miR-126a-5p expression in peripheral blood serum of r*E*g.P29-immunized mice after 1 and 24 weeks by qRT-PCR. **(B)** Anatomical view of protoscolex intraperitoneally infected mice and PBS control mice after 24 weeks. **(C)** Protoscolex isolated from the cyst of a CE patient, magnification 20 × 10. **(D)** Analysis of peripheral blood serum miR-126a-5p expression in protoscolex intraperitoneally infected mice after 1 and 24 weeks by qRT-PCR. **(E)** Determination of serum miR-126a-5p expression in the peripheral blood of CE patients (*n* = 12) and healthy controls (*n* = 16) by qRT-PCR. *P < 0.05, **P < 0.01,***P < 0.001; ns, not significant.

We also explored whether miR-126a-5p could be used as a serum marker for CE patients. The peripheral blood serum of mice immunized with r*E*g.P29 for 1 and 24 weeks was obtained. qRT-PCR experiments were performed to verify the expression of miR-126a-5p in each group. The results showed that 1 week after immunization with r*E*g.P29, serum miR-126a-5p was significantly elevated compared with that in the control group, with no change after 24 weeks of immunization (**Figure 7A**). Notably, in mice infected with protoscolex, peripheral blood serum miR-126a-5p was elevated after 1 week of infection and declined after 24 weeks of infection (**Figures 7B–D**). Similarly, peripheral blood serum miR-126a-5p expression was down in CE patients compared with that in non-CE patients (**Figure 7E**). The result of this cause may be related to the predominance of Th1 response in the early stages of infection and Th2 response in the late stages of infection. miR-126-5p may serve as a serum biomarker for CE patients.

## Discussion

Over the last decade, it has been increasingly demonstrated that vaccination of intermediate hosts is an effective means of controlling the spread of CE (39–41). A large number of vaccines have been developed. The *E*g95, *E*g29, *E*gA31, *E*gG1Y162, and *E*gN123 have shown good immune protection (18, 42–45). Nonetheless, the cost of vaccinating sufficient numbers of animals and the subsequent maintenance may prevent the widespread application of the vaccine. Therefore, exploring the immunological mechanisms by which vaccines work is expected to provide new perspectives for the prevention and treatment of CE. CD4^+^ T cells are considered to be closely related to the infection with *E*g. The Th1-dominated immune response is associated with immune protection, whereas the Th2-dominated immune response is correlated with parasite growth in the late stages of infection (46, 47).

Our team found that r*E*g.29 has good immune protection in sheep and mice (15, 18). Subsequently, we conducted an miRNA microarray analysis of immunized as well as infected mice screened for three miRNAs (miR-126a-5p, miR-378a-5p, miR-1247-5p) with upregulated expression. miR-126a-5p was identified to investigate the potential mechanism of r*E*g.P29 to affect immune changes. It has been reported that miR-126 attenuates Th2 responses by suppressing the asthma phenotype (48). miR-126 also causes SLE by targeting DNA methyltransferase 1 in CD4^+^ T cells (49). Obviously, miR-126 is involved in the development and function of immune cells (50) and can restrain the development of Th2 immune response during leishmaniasis infection (51). Although the expression of IL-4 in mice immunized with r*E*g.P29 was not changed by flow cytometry, which may be the result of the interaction of multiple miRNAs in mice, the intervention of miR-126a-5p alone can cause changes in the expression of IL-4.

A growing evidence base suggests that Notch signaling is involved in CD4^+^ T-cell differentiation and plays a critical regulatory role (52–54). The Notch1 signaling pathway is controversial in the polarization of Th1/Th2 cell subtypes. On the one hand, Notch1 promotes Th1 subtype differentiation during *Helicobacter pylori* infection (29). Taxifolin reduces the proportion of Th1 cells by inhibiting the Notch1 signaling pathway (55). On the other hand, Notch1 signaling promotes the differentiation of Th2 lymphocytes in the airway microenvironment (56). Abnormal activation of Notch1 signaling in B cells promotes Th2 cell-dominated T-cell responses (57). In this study, we first confirmed that the Notch1 signal pathway can promote the differentiation of Th1 cells after 1 week of immunization with r*E*g.P29. It is worth pondering that the DLK1/Notch1 axis does not affect the activation and proliferation of naive CD4^+^ T cells, suggesting that there are other mechanisms affecting activation and proliferation after miR-126a-5p action.

It has been found that hsa-miR-125b-5p may be a promising biomarker for early non-invasive diagnosis of *alveolar echinococcosis* (58). Similarly, miR-126-5p may also serve as a biomarker for early CE infection and as an auxiliary detection molecule for late imaging confirmation. The sequence of mmu‐miR‐126a‐5p and has‐miR‐126‐5p is completely identical (5′CAUUAUUACUUUUGGUACGCG3′), which provides clinical significance to the work we performed in mice. Although the expression of mir-126a-5p increased in the early stage of infection, the expression of miR-126a-5p decreased in the serum of CE mice (24 weeks) and CE patients in the late stage of infection. Due to the early stage of infection, the patients did not have any clinical symptoms. We were unable to determine the early changes in serum hsa-miR-126-5p expression. In addition, the weekly miR-126a-5p assay in r*E*g.P29-immunized and protoscolex-infected mice requires a large number of mice and materials. The above became the limitation of this study.

In summary, we demonstrated that miR-126a-5p enhances the Notch1 signaling pathway by targeting binding to DLK1 and promotes splenic CD4^+^ T-cell differentiation toward Th1 1 week after r*E*g.P29 immunization of mice. miR-126-5p may be a potential promising serum biomarker for CE infection.

## Data Availability Statement

The original contributions presented in the study are included in the article/**Supplementary Material**. Further inquiries can be directed to the corresponding author.

## Ethics Statement

The studies involving human participants were reviewed and approved by the Ethics Committee of Ningxia Medical University. The patients/participants provided their written informed consent to participate in this study. The animal study was reviewed and approved by the Ethics Committee of Ningxia Medical University.

## Author Contributions

XD and MZ jointly conceptualized this experiment. SY and TZ constructed the CE mouse models of *Echinococcus granulosus* infection. JT provided the serum sample from CE patients. CW and YZ assisted in the operation of the experiment. XD completed the first draft and revisions of this manuscript. WZ and MZ offered sufficient financial support for this project. The final version of the manuscript was approved by all authors after careful discussion.

## Funding

This study was funded by the National Natural Science Foundation of China (No. 81860366, No. 32060805, No. 81560335) and Key R&D Projects of Ningxia (No. 2018BEG02003, 2021BEG03088).

## Conflict of Interest

The authors declare that the research was conducted in the absence of any commercial or financial relationships that could be construed as a potential conflict of interest.

## Publisher’s Note

All claims expressed in this article are solely those of the authors and do not necessarily represent those of their affiliated organizations, or those of the publisher, the editors and the reviewers. Any product that may be evaluated in this article, or claim that may be made by its manufacturer, is not guaranteed or endorsed by the publisher.

## Supplementary Material

Supplementary Figure 1 miR-126a-5p promotes the differentiation of naïve CD4^+^ T cells into Th1. **(A) **qRT-PCR assay for optimal concentration of transfected miR-126a-5p mimics in naïve CD4^+^ T cells. **(B)** qRT-PCR assay for optimal concentration of transfected miR-126a-5p inhibitor in naïve CD4^+^ T cells. **P*<0.05, ***P* < 0.01, ****P* < 0.001, *****P* < 0.0001; ns, not significant.

Supplementary Figure 2 DLK1 modulates naïve CD4^+^ T cells differentiation through Notch1 signaling pathway. **(A)** The mRNA expression of DLK1 was detected by qRT-PCR in naïve CD4^+^ T cells transfected with OE-DLK1 or DLK1 siRNA-389,446 and 523 on DLK1. **(B)** Flow assay of the expression of the activation molecules CD25 and CD69 on naïve CD4^+^ T cells transfected with OE-DLK1 or DLK1 siRNA in naïve CD4^+^ T cells. **(C)** CFSE assay in naïve CD4^+^ T cells transfected with OE-DLK1 or DLK1 siRNA on the proliferation of naïve CD4^+^ T. **(D)** qRT-PCR was performed to determine the mRNA expression of HES1 by transfection of DAPT in naïve CD4^+^ T cells. **(E)** Flow assay of the expression of the activation molecules CD25 and CD69 in naïve CD4^+^ T cells by transfection of DAPT in naïve CD4^+^ T cells. **(F)** CFSE assay in naïve CD4^+^ T cells transfected with DAPT on the proliferation of naïve CD4^+^ T cells. ***P *< 0.01, ****P *< 0.001; ns, not significant.
