# Supplementary figures and images for: The Recombinant Eg.P29-Mediated miR-126a-5p Promotes the Differentiation of Mouse Naive CD4+ T Cells via DLK1-Mediated Notch1 Signal Pathway
Source: Front Immunol. 2022 Feb 8;13:773276. doi: 10.3389/fimmu.2022.773276 (PMC8861942; doi:10.3389/fimmu.2022.773276)

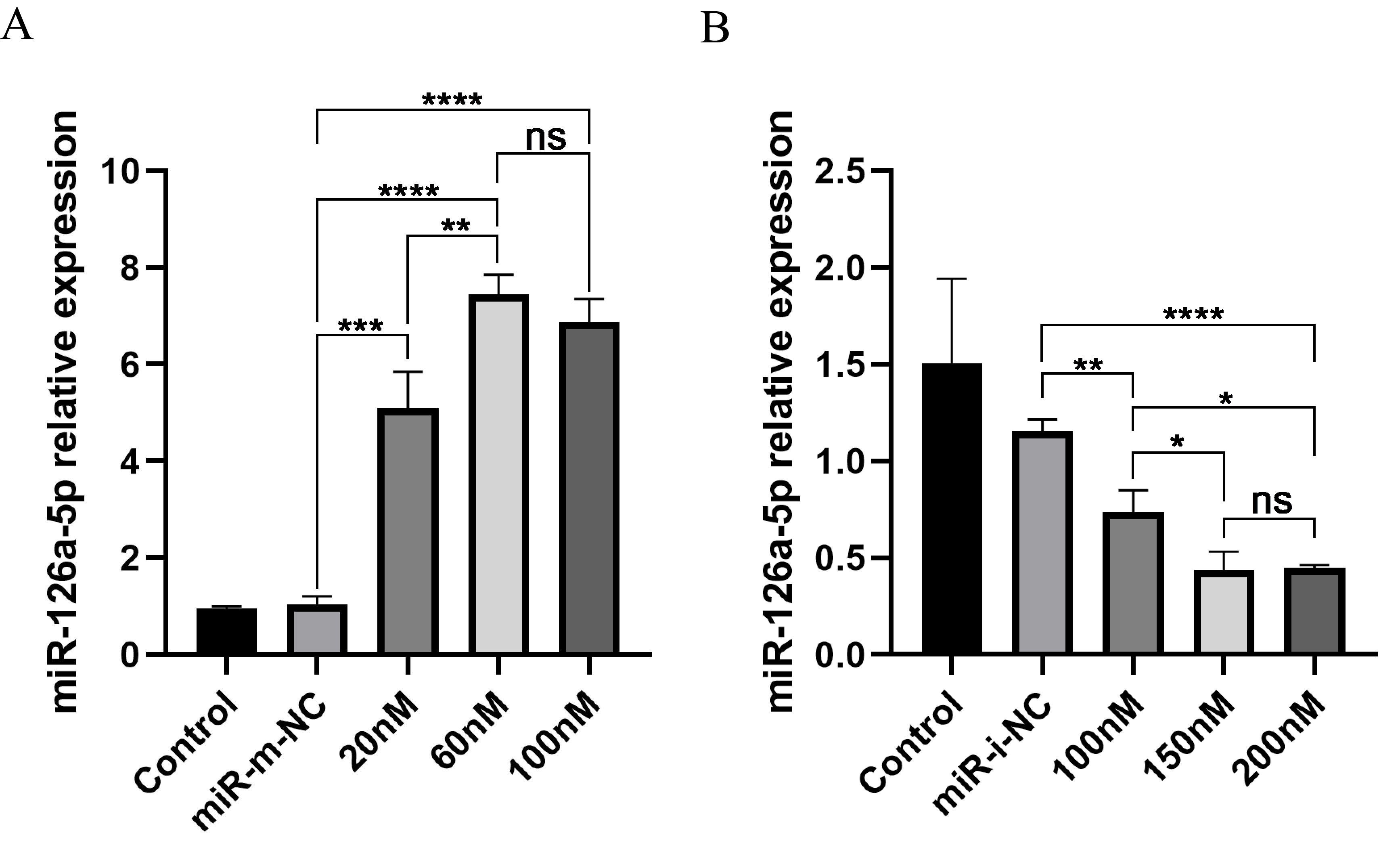

Supplement: Supplementary Figure 1 — miR-126a-5p promotes the differentiation of naïve CD4+ T cells into Th1. (A) qRT-PCR assay for optimal concentration of transfected miR-126a-5p mimics in naïve CD4+ T cells. (B) qRT-PCR assay for optimal concentration of transfected miR-126a-5p inhibitor in naïve CD4+ T cells. *P<0.05, **P < 0.01, ***P < 0.001, ****P < 0.0001; ns, not significant. [file Image_1.tif]
